# Reading and Screen Time: Associations with Behavioral and Sleep Difficulties in Children and Adolescents

**DOI:** 10.3390/pediatric18040107

**Published:** 2026-08-06

**Authors:** Henrike Waterstrat, Nico Grafe, Wieland Kiess, Andreas Merkenschlager, Tanja Poulain

**Affiliations:** 1 LIFE Leipzig Research Center for Civilization Diseases, Leipzig University, 04103 Leipzig, Germany; nico.grafe@medizin.uni-leipzig.de (N.G.); wieland.kiess@medizin.uni-leipzig.de (W.K.); tanja.poulain@medizin.uni-leipzig.de (T.P.); 2 Department of Women and Child Health, Hospital for Children and Adolescents and Center for Pediatric Research (CPL), Leipzig University, 04103 Leipzig, Germany; andreas.merkenschlager@medizin.uni-leipzig.de; 3 German Center for Child and Adolescent Health (DZKJ), Partner Site Leipzig/Dresden, 04103 Leipzig, Germany

**Keywords:** screen time, reading, sleep difficulties, behavioral difficulties, children

## Abstract

Objective: This study examined reading behavior and screen time in children and adolescents and their associations with sleep behavior and behavioral difficulties. Methods: This study was conducted as part of the LIFE Child study (Germany). Participants were 579 6- to 10.5-year-old children (younger age group) and 972 10.5- to 18-year-old children and adolescents (older age group). Information on reading (duration of reading, on paper or electronically), screen time, sleep difficulties, and behavioral difficulties was assessed via parental (younger sample) or self-reported (older sample) questionnaires. Associations of reading behavior and screen time with child age, sex, maternal education, behavioral difficulties, and sleep difficulties were assessed using linear regression analyses. Results: Girls reported longer reading times than boys, whereas boys reported longer daily screen time. In the younger age group, reading time increased with age. Daily screen time increased with age in both age groups. Higher screen time was significantly associated with more sleep difficulties in both age groups and with more behavioral difficulties in the older age group. In the younger age group, reading time was not associated with screen time, sleep difficulties, or behavioral difficulties. In the older age group, however, longer reading times were significantly associated with longer screen time, more behavioral difficulties, and more problematic sleep. Conclusions: These results indicate that both the use of electronic media and reading behavior are relevant for understanding child health and development. The findings suggest that guidance for families of older children should address not only screen use but also balance and context of reading activities.

## 1. Introduction

Reading is a fundamental skill that enables active participation in society and plays a central role in educational development and daily life. In children and adolescents, reading engagement has been associated with enhanced vocabulary acquisition [1], greater interest in literature [2], and improved language and cognitive abilities [3,4]. Beyond its academic benefits, emerging evidence suggests that reading habits may also influence emotional well-being [4], sleep quality [5,6], and the establishment of healthy daily routines [7], thereby underscoring their potential relevance from a public health and developmental perspective.

Representative survey data from Germany in 2024 indicate that daily reading is practiced by only 13% of girls and 7% of boys aged 7 to 13 years, while approximately 16% of children in this age group report no reading at all [8]. Among adolescents aged 12 to 19 years, the proportion of daily or at least weekly readers is higher, with 47% of girls and 28% of boys engaging in regular reading [9]. These figures highlight considerable age- and sex-related disparities in reading behavior and raise the question of how both reading and screen time relate to developmental and health outcomes. Another aspect of children’s everyday life is the time they spend in front of electronic screens. In 2024, more than 92% of German children aged 6 to 13 watched television at least once a week, and 42% used TikTok daily or more than once a week [8]. For older children aged 12 to 19 years, the average time spent on the internet each day was 201 min in 2024 [9]. In contrast to reading, the intensive use of electronic media has been shown to be associated with poorer wellbeing [10], more behavioral difficulties [11], and poorer sleep outcomes [12,13], although findings remain inconsistent [14].

Previous research suggests that reading and screen use differ according to age, sex, and socioeconomic background [14]. Girls were shown to report higher reading motivation and engage in reading more frequently than boys, with these differences often increasing during childhood and adolescence [15,16]. In contrast, boys were shown to report greater engagement in screen-based activities, particularly gaming and other entertainment-related media use [17]. Socioeconomic status (SES) has also been associated with reading behavior and screen time, with children from higher-SES families generally reporting more frequent reading [16] but less frequent use of electronic media [18].

Despite the growing body of research on children’s reading and their use of electronic media, several knowledge gaps remain. First, most studies have focused on screen time or reading behavior separately, whereas their interrelationship has received less attention [14]. Second, although screen time has been linked to sleep and behavioral outcomes [18,19], the potential role of reading as a protective factor against these difficulties remains insufficiently understood [10], particularly in children and adolescents. Finally, evidence on sociodemographic differences in reading and screen use patterns is still limited in contemporary populations exposed to rapidly evolving digital media environments. Therefore, the present study aimed to examine the associations of reading and screen time with sociodemographic characteristics as well as sleep and behavioral difficulties in a large sample of children and adolescents.

Based on previous findings, we hypothesized that the older children get, the more they read and the more time they spend in front of screens. At the same time, we hypothesized that boys spend more time in front of screens than girls, and girls read more frequently than boys. Furthermore, we hypothesized that children from families with low socioeconomic status (SES) spend more time in front of screens and less time reading. Finally, we expected that children who spend a lot of time in front of screens have more sleep problems and behavioral difficulties, whereas these difficulties may be less common in children who spend more time reading. By identifying sociodemographic differences and behavioral correlates of reading and screen use, this study may help inform targeted prevention and health promotion strategies, particularly for vulnerable families and children at increased risk for behavioral or sleep-related difficulties.

## 2. Materials and Methods

### 2.1. Participants

Data were collected between 2021 and 2024 within the LIFE Child Study, a cohort study conducted in the city of Leipzig, Germany. The LIFE Child study assesses the typical development of healthy children and the development of non-communicable diseases, e.g., obesity, depression, or allergies. All children not suffering from any chromosomal or syndromic diseases are eligible for participation. They are recruited between birth and 16 years of age and mainly come from the city and surrounding areas of Leipzig, a large city in the East of Germany.

Until the age of 21 years, all participants were invited to attend follow-up visits once per year [20,21].

The study program includes medical examinations and tests as well as questionnaires on children’s and adolescents’ health and behavior. Before participation, all parents provided informed written consent.

### 2.2. Study Population

For the present project, all children and adolescents aged 6 to 18 years who (or whose parents) had completed questionnaires on reading, screen time, sleep difficulties, and behavioral difficulties were eligible (*n* = 1551).

Participants were divided into a younger (6–10 years) and an older age group (11–17 years). This categorization was chosen because age 11 typically marks the transition from primary to secondary school in Germany and, more importantly, because different age-appropriate assessment instruments were used: parent-reported questionnaires for younger children and self-reported questionnaires for older children and adolescents.

### 2.3. Reading and Screen Time

For the daily duration of reading, children (older age group) or their parents (younger age group) were asked to indicate the time they read on a typical weekday and a weekend day (excluding reading at or for school). Importantly, because children may read both printed and electronic books (e.g., using e-book readers), participants were asked to report time spent reading on paper as well as electronically.

Participants could choose between 5 response options (not at all, approximately 30 min per day, approximately 1–2 h per day, approximately 3–4 h per day, more than 4 h per day). The answers were transformed to durations (0, 0.5, 1.5, 3.5 and 5). Durations on weekdays and weekends were considered separately but also combined to one variable (weighted mean = (duration on weekday × 5 + duration on weekend × 2)/7).

Regarding screen time, children or their parents specified their screen time (including time on mobile phone, television, computer, tablet, excluding screen time at or for school) on a typical weekday and on weekends, with up to half-hour accuracy. Durations on weekdays and weekends were considered separately but also combined to one variable (weighted mean = (screen time on weekday × 5 + screen time on weekend × 2)/7).

### 2.4. Behavioral Difficulties

In order to assess behavioral difficulties, the children or their parents completed the Strengths and Difficulties Questionnaire (SDQ). The SDQ comprises 25 questions on five scales (emotional problems, conduct problems, hyperactivity/inattention, peer relationship problems, and prosocial behavior) [22]. Each item is answered on a 3-point Likert scale. Items of the same scale (5 items per scale) are combined to scores ranging from 0 to 10, with higher scores indicating more difficulties or more prosocial behavior. The scores on emotional problems and peer-relationship problems were summed to an internalizing problems score (ranging from 0 to 20), while the scores on conduct problems and hyperactivity/inattention were combined to an externalizing problems score (ranging from 0 to 20). Finally, all scale values except prosocial behavior were combined to a total difficulties score ranging from 0 to 40.

### 2.5. Sleep Difficulties

In the younger age group, parents completed the Children’s Sleep Habits Questionnaire (CSHQ) [23,24]. This questionnaire consists of 33 items that are combined to 8 sub-scores (sleep anxiety, night wakings, daytime sleepiness, sleep duration, sleep-disordered breathing, parasomnias, bedtime resistance, and sleep onset delay) and a total sleep difficulties score. Each item can be answered on a 3-point Likert scale. The total sleep difficulties score, ranging from 33 to 99, was analyzed in this study. In a representative German sample, a total score of 47 corresponded to the 90th percentile [23]. Accordingly, scores exceeding this threshold were considered indicative of problematic levels of sleep-related difficulties. In the older age group, children completed a short version of the Sleep Self Report (SSR) [25,26] consisting of 8 items answered on a 3-point Likert scale (sleep latency, regularity of bedtimes, nighttime awakening, problems falling back asleep after waking up, nightmares, difficulty waking up in the morning, daytime sleepiness, daytime naps). The item responses were combined to a total sleep difficulties score ranging from 8 to 24. This score was analyzed in this study.

### 2.6. Socioeconomic Status

A family’s SES was estimated by mothers’ education. Parents were asked to indicate the highest school degree and the highest professional education of the child’s mother. Information on both was combined to a score ranging from 1 to 7, with higher scores indicating higher education [27].

### 2.7. Data Analysis

The data analysis was performed using R (version 4.3.2, R Foundation for Statistical Computing, Vienna, Austria). Statistical comparisons between the younger and the older age groups were conducted using independent-samples *t*-tests. Associations between reading time or screen time (dependent variables) and age, sex, and maternal education (independent variables) were assessed using linear regression analyses. Linear regression analyses were also applied to investigate the associations between reading and screen time (independent variables) and sleep difficulties as well as behavioral difficulties (dependent variables). Reading and screen time were included simultaneously, adjusting for age, sex, and maternal education.

To account for multiple comparisons, the false discovery rate (FDR) was controlled using the Benjamini–Hochberg correction.

## 3. Results

Characteristics of children in the younger (*n* = 579) and the older (*n* = 972) age group are shown in Table 1.

The analyses revealed significantly longer reading times, longer screen time, and more behavioral difficulties in the older than in the younger age group (all *p* < 0.001). The maternal education score, in contrast, did not differ significantly between both age groups (*p* = 0.168).

The CSHQ screening revealed that 26% of the children in the younger age group showed elevated sleep problems above the cut-off score of 47. The means and standard deviations of the single CSHQ scores are shown in Supplementary Table S1.

**Figure 1:**
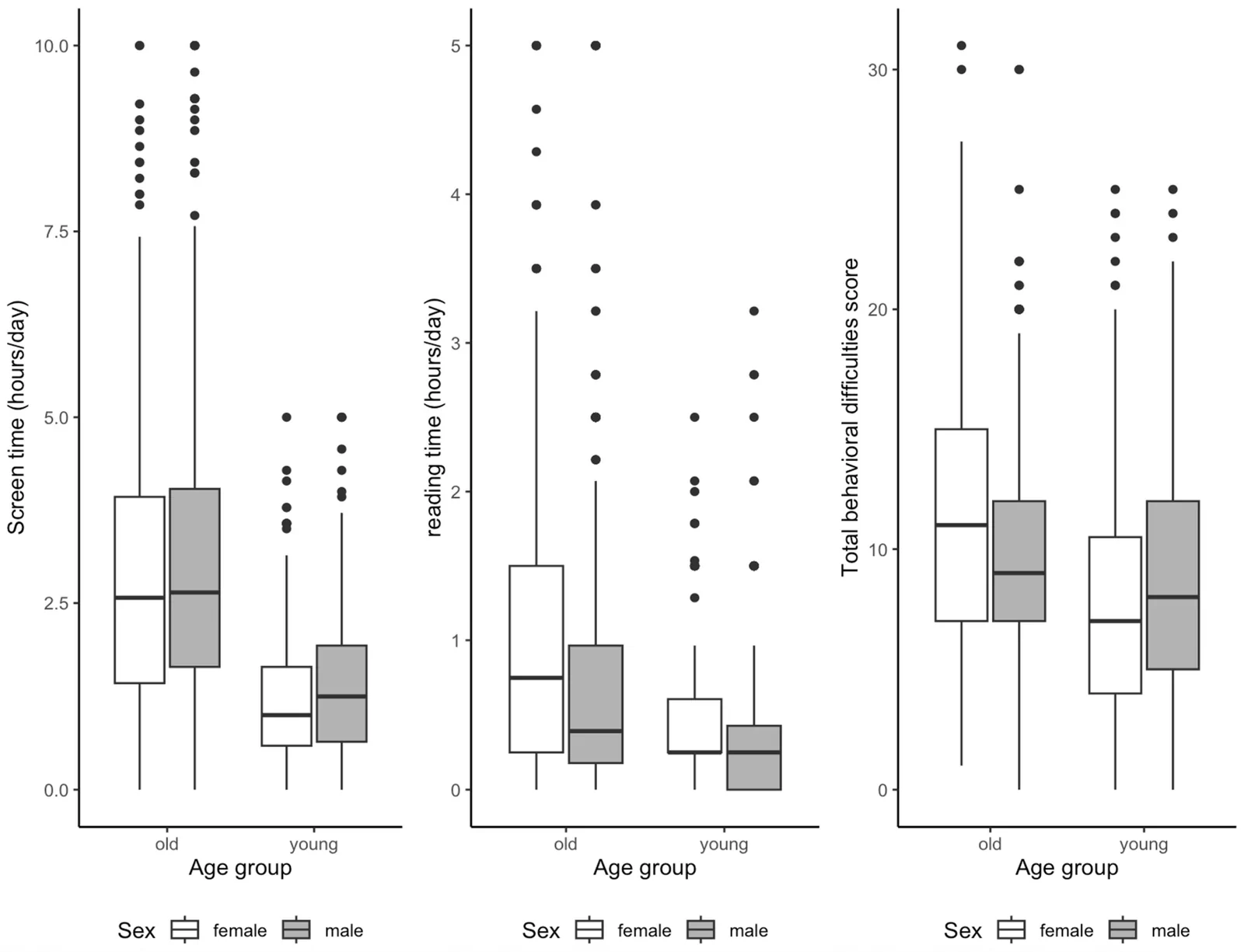
Reading time, screen time and behavioral difficulties by age group and sex.

Figure 1 shows reading times, screen time, and total behavioral difficulties scores, stratified by age group and sex.

### 3.1. Association Between Reading, Screen Time, and Sociodemographic Characteristics

The associations between reading behavior and screen time (dependent variables) and age, sex, and SES (independent variables) are shown in Table 2. Regarding reading, we observed a significant positive association with age in the younger age group (b = 0.09, 95% CI [0.06, 0.12]; *p* < 0.001). Children aged 6 years read for an average of around 0.21 h a day and this duration rose to around 0.56 h a day for children aged 10. In the older age group, in contrast, we observed no association with age (see Table 2). A sex difference, with significantly higher reading durations in girls (estimated mean = 0.92 h) than boys (estimated mean = 0.71 h), was shown in the older age group (b = −0.22, 95% CI [−0.34, −0.10]; *p* = 0.001) but not in the younger age group (see Table 2).

Maternal education was not associated with reading.

Regarding screen time, the analyses revealed significant positive associations with age in both age groups (younger age group: b = 0.25; 95% CI [0.19, 0.30]; *p* < 0.001; older age group: b = 0.38; 95% CI [0.32, 0.42]; *p* < 0.001). The 10-year-olds already averaged 1.8 h per day, while the 18-year-olds spent on average 4.8 h per day using screens.

In the younger age group, but not in the older age group, boys (estimated mean = 1.41 h) spent more time in front of the screen than girls (estimated mean = 1.24 h).

The analyses also revealed a significant negative association between screen time and maternal education in the older, but not in the younger age group. The higher the SES, the less time the children spent in front of screens (b = −0.14; 95% CI [−0.21, −0.07]; *p* < 0.001).

### 3.2. Associations Between Reading and Screen Time

In the younger age group, we observed no significant association between screen time and reading (b = −0.04, 95% CI [−0.08, 0.003]; *p* = 0.11).

In the older age group, in contrast, we observed a significant positive association between reading and screen time (b = 0.05, 95% CI [0.01, 0.08]; *p* = 0.02). Children and adolescents who, on average, spent two hours a day in front of the screen were estimated to read for an average of 0.76 h a day. In contrast, children who spent 5 h a day in front of a screen were estimated to spend around 0.90 h a day reading.

### 3.3. Associations of Reading and Screen Time with Sleep and Behavioral Difficulties

Table 3 shows the associations of reading behavior and screen time with sleep and behavioral difficulties.

In the younger age group, higher screen time was associated with significantly more sleep difficulties (b = 0.75, 95% CI [0.19, 1.31]; *p* = 0.021) but not with more behavioral difficulties (Table 3). Children with about one hour of daily screen use had an average total sleep difficulties score of 43, while those with two hours of screen time had an average score of 44. Regarding the single CSHQ subscales, screen time was significantly associated with sleep anxiety (b = 0.23; 95% CI [0.08, 0.39]; *p* = 0.01), but not with the other subscales (Supplementary Table S1).

In contrast, the analyses revealed no significant association between reading and sleep or behavioral difficulties in the younger age group (Table 3). In the older age group, we observed significant positive associations between reading time and sleep difficulties (b = 0.39, 95% CI [0.16, 0.63]; *p* = 0.003). Children who read for approximately half an hour per day had an average sleep difficulties score of 12.44, while those who read for approximately two hours per day had an average score of 13.03.

Reading was also significantly associated with more behavioral difficulties (b = 0.53; 95% CI [0.16, 0.90]; *p* = 0.015). The estimated total difficulties score, on average, was 10.6 in children who read 1 h per day, but 11.06 in children who read about 2 h each day. When distinguishing internalizing and externalizing behavioral difficulties, however, only the association with internalizing behavioral difficulties was significant (b = 0.38; 95% CI [0.16, 0.60]; *p* = 0.002).

As in the younger age group, screen time was significantly associated with more sleep difficulties in the older age group (b = 0.22; 95% CI [0.09, 0.35]; *p* = 0.004). In addition, the analyses also revealed a significant association between screen time and more behavioral difficulties (b = 0.52; 95% CI [0.33, 0.72]; *p* < 0.001). Children who spent an average of 2 h a day in front of a screen had a total sleep difficulties score of approximately 12 points and a total behavioral difficulties score of 9.94. In contrast, children who spent 8 h a day in front of a screen had a score of approximately 14 points (sleep) or 12.90 points (behavioral difficulties). Regarding behavioral difficulties, both internalizing (b = 0.24; 95% CI [0.12, 0.37]; *p* < 0.001) and externalizing (b = 0.28; 95% CI [0.17, 0.40]; *p* < 0.001) behavioral difficulties were significantly associated with screen time.

### 3.4. Differences Between Weekday and Weekend Reading and Screen Time

All analyses examining the associations between reading or screen time and sleep or behavioral difficulties were additionally conducted separately for weekday and weekend reading/screen time. The results were highly similar across weekdays and weekends and closely mirrored those obtained using the combined measure reported above.

## 4. Discussion

Given the growing digitalization of children’s everyday lives, understanding the relationship between reading and screen use and their associations with behavioral and sleep difficulties at different ages throughout development is of increasing public health relevance. Previous research suggests that reading may promote cognitive and psychosocial development [1,3,4,18], whereas excessive screen time has been associated with sleep disturbances and behavioral difficulties [10,11,12,17].

It is important to mention that the children participating in the present study came from well-educated households and that disadvantaged families were underrepresented in our study.

The screen time and reading duration in our sample were broadly comparable with durations shown in previous studies [13,24]. The behavioral difficulties scores [28] as well as the sleep difficulties scores in the younger age group were also comparable with previous findings [20].

Sleep difficulties in the older age group were assessed differently and, therefore, should not be compared with previous studies. Nevertheless, the observed associations between media use, reading behavior, and behavioral or sleep difficulties may still be clinically relevant, as they point toward modifiable everyday behaviors that could be addressed in pediatric prevention, health promotion, and family counseling settings.

### 4.1. Associations Between Screen Time/Reading and Sex

Regarding reading, we only observed a significant sex difference in the older age group, with girls reading more than boys. In contrast, boys in the older age group spent more time using electronic media than girls. No sex differences were observed in the younger age group. These findings suggest that girls might be more interested in reading (printed books or on screens), at least during adolescence, while adolescent boys might be more interested in electronic media. This assumption is in line with other studies highlighting a particularly higher interest of boys (versus girls) in playing video games [8,9]. Previous studies also showed a higher interest in reading in girls than boys [8,9].

### 4.2. Associations Between Screen Time/Reading and Age

Regarding reading, different patterns emerged across the age groups. In the younger age group, reading duration increased with growing age. This change can be explained by changing interests and increasing autonomy with increasing age. A similar result was already shown in previous studies [1]. In young children, the impulse to read often comes from the parents and the motivation to read has to be developed by the children over the years [7].

These findings underline the importance of supporting reading habits early in life through parental involvement and family routines, as such habits may contribute not only to educational development but also to healthy daily structures and bedtime routines.

In terms of screen time, an increase in daily screen time with increasing age was observed in both age groups. This may be because children are learning to navigate the internet independently and are developing more interests over the years, e.g., using social media or video games, all of which are associated with an increase in screen time [8,9]. In addition, parents’ efforts and ability to restrict the use of electronic media often diminishes as children grow older [29].

### 4.3. Associations Between Screen Time/Reading and Socioeconomic Status

The analyses revealed no significant associations between maternal education and the time spent reading, contrasting with findings from recent studies [30]. This may be due to the sample mainly comprising families with a middle or high SES, making such associations harder to detect.

However, children from families with a higher SES spent less time in front of screens. This association was only significant in the older age group but pointed in the same direction in the younger age group. In line with previous studies [30,31], this suggests that less educated parents may be less aware of potentially detrimental effects of high use of electronic media or regulate their children’s media use less strictly. This effect may still become apparent even in samples of predominantly middle and high SES. These findings may be relevant for public health interventions, as families with lower SES could particularly benefit from accessible information and practical guidance regarding healthy media habits and the potential behavioral consequences of excessive screen exposure.

### 4.4. Associations Between Screen Time and Reading

The association between screen time and reading differed between the two age groups. In the younger age group, screen time was not associated with reading. In the older age group, we observed a positive association between screen time and reading, i.e., children who spent more time in front of screens also spent more time reading. Older children and adolescents may enjoy immersing themselves in other worlds both through reading as well as through the use of electronic media. Importantly, we did not differentiate reading on paper or on a screen (e.g., an e-book reader). Therefore, the positive association between reading and screen time in older children might also indicate that some children are attracted by all screen activities, including electronic reading, gaming, or streaming. This distinction may be important for future prevention strategies and family recommendations, as reading on screens and traditional reading may differ substantially in their associations with sleep and behavioral outcomes.

### 4.5. Association Between Reading/Screen Time and Sleep and Behavioral Difficulties

The associations between screen time and reading, on the one hand, and behavioral and sleep difficulties, on the other, differed between age groups. In the younger age group, children spending more time using electronic media had more sleep difficulties. This might be related to evening distraction or the circadian rhythm shifts due to light exposure [11,12]. However, we did not show significant associations between screen time and behavioral difficulties. Also, reading was not related to sleep or behavioral difficulties.

In the older age group, in contrast, both reading and screen time were associated with significantly more behavioral difficulties and more sleep difficulties, i.e., children and adolescents who spent more time reading (on paper or on screens) or using electronic media reported more behavioral and also more sleep difficulties. However, while longer screen time was associated with both internalizing as well as externalizing behavioral difficulties, reading was only associated with more internalizing problems.

Regarding screen time, associations with behavioral and sleep difficulties have been shown in several previous studies [10,13,32] and, therefore, are not surprising. He et al. [33] showed in their meta-analysis that greater screen time increases the risk of poor sleep outcomes, with each additional daily hour linked to shorter sleep and more disturbances. Our findings supports the assumption that screen exposure may negatively affect sleep through mechanisms such as delayed bedtimes, increased physiological arousal, or light-induced suppression of melatonin secretion [32,33,34].

However, based on previous studies [10,13], we did not expect reading time to be associated with more behavioral or sleep difficulties. In fact, regular reading has often been linked to positive outcomes, including better mental health, greater well-being, and higher quality of life [35]. Therefore, the positive association between reading and sleep difficulties observed in our sample appears somewhat unexpected. The association between reading and internalizing difficulties, however, may be interpreted differently. Previous research has suggested that children and adolescents with higher levels of anxiety, emotional distress, or other internalizing symptoms may spend more time engaging in solitary and cognitively absorbing activities such as reading [36]. In this context, reading may serve as a coping strategy, a form of emotion regulation, or a means of temporary withdrawal from stressful situations. Thus, the observed association may not necessarily indicate that reading contributes to internalizing difficulties; rather, young people experiencing emotional problems may be more likely to engage in reading.

This interpretation may also help explain why both reading and screen time were positively associated with behavioral and sleep difficulties in the older age group. Although reading and electronic media use are often regarded as fundamentally different activities, both may reflect underlying psychosocial processes. Children and adolescents experiencing emotional distress, psychosocial stress, or sleep problems may increasingly turn to either reading or screen-based activities as accessible forms of coping, distraction, or escape. At the same time, extensive engagement in either activity could reduce opportunities for social interaction, delay bedtimes, or contribute to insufficient sleep, thereby further reinforcing behavioral and sleep difficulties [36].

However, an important methodological limitation is that reading time may have included both paper-based and electronic reading, whereas screen time captured the use of electronic devices. Thus, electronic reading may have been reflected in both variables, potentially contributing to the similar associations observed for reading and screen time in the older age group. Another limitation is that sleep and behavioral difficulties were assessed using different informants and, regarding sleep, different instruments across age groups. Consequently, observed differences in associations between younger and older children may not solely reflect developmental changes but may also have been influenced by methodological differences in assessment procedures.

### 4.6. Strengths and Limitations

This study has several methodological strengths. These include the large, population-based sample spanning two distinct developmental periods, the simultaneous assessment of both reading behavior and screen time within the same cohort, and the use of well-validated instruments to assess sleep and behavioral difficulties. The age-stratified design further allowed for direct comparisons of these associations across childhood and adolescence, providing valuable insights into developmental differences.

At the same time, some limitations should be considered when interpreting the findings. First, parental reports—while practical and widely used—may be subject to social desirability or recall biases. Second, as reading, as measured in this study, can occur both on paper and on screens, some children may have inadvertently counted electronic reading as both “reading” and “screen time,” potentially confounding the associations between these variables. Third, the assessment of sleep difficulties differed between age groups, with parent-reported CSHQ data used for younger children and self-reported SSR data used for older children and adolescents. Given these differences in both measurement instruments and respondents, direct comparisons of sleep difficulties across age groups are limited.

Fourth, screen time was assessed as a global measure without differentiating between specific activities (e.g., entertainment, educational, social) or the timing of use. However, recent longitudinal evidence suggests that evening use and entertainment-oriented activities may be particularly detrimental for adolescent sleep [37]. Fifth, the sample composition in terms of socioeconomic status (SES) should be taken into account. If there had been more families with lower SES in the cohorts, the results would likely have shown higher screen time and behavioral problems. However, the strength of the associations would likely have increased rather than decreased [38].

Despite these limitations, the study adds important evidence to the growing literature on media use and child development, highlighting the need for differentiated approaches in future research and family guidance.

## 5. Conclusions

Reading and the use of electronic media can be clearly distinguished from one another in preschool and elementary school age children and show different associations with sleep and behavioral problems at that age. In adolescence, however, the frequency of reading on paper or screens and the use of electronic media are significantly associated and both show associations with sleep and behavioral difficulties. These results provide evidence that not only intensive use of electronic media but also intensive reading, which may actually take place on screens, should be taken seriously in children and adolescents and could indicate or lead to psychosocial problems. However, further studies should investigate the extent to which the same associations occur depending on whether children and adolescents read on paper or electronically.

## Figures and Tables

**Table 1:** Distribution of the recorded variables among the participating children (*n* = 1551), broken down by age group and analyzed for statistical comparisons.

		Younger Age Group (*n* = 579)	Older Age Group (*n* = 972)	*p*-Value by *t*-Test	Cohen’s *d*
Sex	*n* (%) female *n* (%) male	270 (47%) 309 (53%)	461 (47%) 511 (53%)		
Age	Mean (sd) years	8.10 (1.33)	13.03 (2.06)		
Reading	Mean (sd) hours/day	0.39 (0.46)	0.80 (0.95)	*p* < 0.001	0.54
Reading during the week	Mean (sd) hours/day	0.37 (0.43)	0.76 (0.94)	*p* < 0.001	0.54
Reading at the weekend	Mean (sd) hours/day	0.46 (0.59)	0.92 (1.10)	*p* < 0.001	0.50
Screen time	Mean (sd) hours/day	1.33 (0.96)	2.97 (1.92)	*p* < 0.001	1.03
Screen time during the week	Mean (sd) hours/day	1.08 (0.92)	2.70 (1.91)	*p* < 0.001	1.05
Screen time at the weekend	Mean (sd) hours/day	1.99 (1.35)	3.69 (2.52)	*p* < 0.001	0.82
Total sleep difficulties score *	Mean (sd)	43.48 (6.17)	12.57 (2.56)		
Total behavioral difficulties score	Mean (sd)	8.39 (5.26)	10.44 (5.05)	*p* < 0.001	0.40
Internalizing behavioral difficulties score	Mean (sd)	2.85 (2.68)	4.82 (3.12)	*p* < 0.001	0.66
Externalizing behavioral difficulties score	Mean (sd)	5.54 (3.66)	5.60 (3.06)	*p* = 0.874	0.02
Maternal education score	Mean (sd)	5.72 (1.44)	5.59 (1.48)	*p* = 0.168	0.09

* CSHQ total sleep difficulties score in younger age group (possible range 33–99); SSR total sleep difficulties score in older age group (possible range 8–24).

**Table 2:** Associations between reading behavior, screen time, and socio-demographic characteristics.

		Reading	Screen Time
Younger age group			
Age	b [95% CI]	0.09 [0.06, 0.12]	0.25 [0.19, 0.30]
	*p*	*p* < 0.001	*p* < 0.001
Sex (male)	b [95% CI]	−0.03 [−0.10, 0.04]	0.17 [0.03, 0.32]
	*p*	0.563	0.045
Maternal education	b [95% CI]	−0.002 [−0.03, 0.02]	−0.05 [−0.11, −0.004]
	*p*	0.931	0.072
Older age group			
Age	b [95% CI]	−0.005 [−0.03, 0.02]	0.38 [0.32, 0.42]
	*p*	*p* = 0.856	*p* < 0.001
Sex (male)	b [95% CI]	−0.22 [−0.34, −0.10]	0.24 [0.02, 0.46]
	*p*	*p* = 0.001	0.069
Maternal education	b [95% CI]	0.03 [−0.01, 0.07]	−0.14 [−0.21, −0.07]
	*p*	0.209	*p* < 0.001

**Table 3:** Associations of reading behavior and screen time with sleep and behavioral difficulties.

		Sleep Difficulties	Behavioral Difficulties	Internalizing Behavioral Difficulties	Externalizing Behavioral Difficulties
Younger age group					
Reading	b [95% CI]	0.03 [−1.10, 1.16]	−0.38 [−1.41, 0.65]	−0.23 [−0.76, 0.30]	−0.15 [−0.86, 0.56]
	*p*	0.958	0.604	0.558	0.838
Screen time	b [95% CI]	0.75 [0.19, 1.31]	0.21 [−0.30, 0.72]	0.20 [−0.06, 0.46]	−0.01 [−0.36, 0.34]
	*p*	0.021	0.604	0.234	0.958
Older age group					
Reading	b [95% CI]	0.39 [0.16, 0.63]	0.53 [0.16, 0.90]	0.38 [0.16, 0.60]	0.15 [−0.78, 0.37]
	*p*	0.003	0.015	*p* = 0.002	0.314
Screen time	b [95% CI]	0.22 [0.09, 0.35]	0.52 [0.33, 0.72]	0.24 [0.12, 0.37]	0.28 [0.17, 0.40]
	*p*	0.004	*p* < 0.001	*p* < 0.001	*p* < 0.001

All associations are adjusted for age, sex, and maternal education.

## Data Availability

Due to legal restrictions and the informed consent provided by the participants, the dataset cannot be made publicly available. Researchers interested in accessing the data may contact the Research Data Management Office of the Medical Faculty, Leipzig University, at forschungsdaten@medizin.uni-leipzig.de for further information. The dataset ID is PV786.

## Supplementary Materials

The following supporting information can be downloaded at: https://www.mdpi.com/article/10.3390/pediatric18040107/s1, Table S1: CSHQ subscale scores: means and standard deviations (younger cohort, *n* = 579); Table S2: Associations of CSHQ subscales with reading time, screen time, and behavioral difficulties in the younger age group (6–10 years, *n* = 579).
