# Supplementary material for: Reading and Screen Time: Associations with Behavioral and Sleep Difficulties in Children and Adolescents
Source: Pediatr Rep. 2026 Aug 6;18(4):107. doi: 10.3390/pediatric18040107 (PMC13516216; doi:10.3390/pediatric18040107)
Supplement: Supplementary file 1 [file pediatrrep-18-00107-s001.zip › pediatrrep-4343757-supplementary.pdf]

Supplements

Supplementary Table S1. CSHQ subscale scores: means and standard deviations (younger age group, n = 579).

CSHQ Scales		
Total sleep disturbance score	Mean (sd)	43.48 (6.17)
Sleep anxiety	Mean (sd)	5.22 (1.75)
Night wakings	Mean (sd)	3.57 (0.98)
Daytime sleepiness	Mean (sd)	12.67 (2.93)
Sleep duration	Mean (sd)	3.83 (1.26)
Sleep disordered breathing	Mean (sd)	3.22 (0.55)
Parasomnias	Mean (sd)	8.23 (1.42)
Bedtime resistance	Mean (sd)	7.88 (2.51)
Sleep onset delay	Mean (sd)	1.53 (0.70)

Supplementary Table S2. Associations of reading time and screen time with the single CSHQ subscales in the younger age group (6–10 years, n = 579).

		Reading	Screen time
Total sleep disturbance score	b [95% CI]<br>p	0.03 [-1.10, 1.16]<br>p = 0.958	0.75 [0.19, 1.31]<br>p = 0.021
Sleep anxiety	b [95% CI]<br>p	-0.12 [-0.44, 0.20]<br>p = 0.601	0.23 [0.08, 0.39]<br>p = 0.001
Night wakings	b [95% CI]<br>p	0.01 [-0.16, 0.19]<br>p = 0.931	0.07 [-0.01, 0.16]<br>p = 0.173
Daytime sleepiness	b [95% CI]<br>p	0.10 [-0.44, 0.64]<br>p = 0.856	0.11 [-0.16, 0.38]<br>p = 0.558
Sleep duration	b [95% CI]<br>p	-0.22 [-0.45, 0.01]<br>p = 0.115	0.09 [-0.02, 0.21]<br>p = 0.209
Sleep disordered breathing	b [95% CI]<br>p	0.004 [-0.09, 0.10]<br>p = 0.958	0.04 [-0.01, 0.09]<br>p = 0.169
Parasomnias	b [95% CI]<br>p	0.12 [-0.14, 0.38]<br>p = 0.532	0.05 [-0.07, 0.18]<br>p = 0.521
Bedtime resistance	b [95% CI]<br>p	0.06 [-0.39, 0.52]<br>p = 0.899	0.26 [0.03, 0.50]<br>p = 0.079
Sleep onset delay	b [95% CI]<br>p	0.01 [-0.16, 0.14]<br>p = 0.923	0.02 [-0.04, 0.09]<br>p = 0.636
